# The metabolic role of PFKFB4 in androgen-independent growth in vitro and PFKFB4 expression in human prostate cancer tissue

**DOI:** 10.1186/s12894-020-00635-0

**Published:** 2020-06-01

**Authors:** Ximing Li, Zhi Chen, Zuan Li, Guihai Huang, Junhao Lin, Qiang Wei, Jianbo Liang, Wei Li

**Affiliations:** 1 Department of Urology, The People’s Hospital of Guangxi Zhuang Autonomous Region, Nanning, China; 2 Graduate School, Guangxi Medical University, Nanning, China; 3 Department of Pathology, The People’s Hospital of Guangxi Zhuang Autonomous Region, Nanning, China

**Keywords:** Prostate cancer, PFKFB4, CRPC, LNCaP cells, Metabolism

## Abstract

**Background:**

It is well known that androgen-deprivation therapy (ADT) can inevitably drive prostate cancer (PCa) cells into a castration-resistant state. According to the “Warburg effect”, the metabolism of aggressive tumor cells increases significantly. The growth of cancer cells depends on glycolysis, which may be a potential target for cancer control. 6-Phosphofructo-2-kinase/fructose-2,6-biphosphatase 4 (PFKFB4) plays key roles in the proliferation and metastasis of PCa cells. However, there is very limited knowledge on the role of PFKFB4 in the conversion to castration resistance. The present study aimed to determine the changes in glucose consumption and PFKFB4 expression in LNCaP cells and androgen-independent LNCaP (LNCaP-AI) cells during the whole process of androgen-independent growth. Additionally, PFKFB4 expression in human PCa tissues was evaluated.

**Methods:**

We established an androgen-independent LNCaP-AI cell line derived from LNCaP cells to mimic the traits of castration resistance in vitro. LNCaP-AI and LNCaP cells were cultured in the corresponding medium containing the same amount of glucose. At the end of experiments, the medium supernatant and blank medium were collected, and absorbance was measured. LNCaP-AI and LNCaP cells were harvested to detect PFKFB4 expression by Western blotting. Prostate tissue samples including PCa tissue, carcinoma-adjacent tissue and benign prostatic hyperplasia (BPH) tissue specimens were evaluated for PFKFB4 expression using immunohistochemistry.

**Results:**

In 18 h supernatant samples, the glucose consumption and lactate secretion of LNCaP-AI cells were higher than those of LNCaP cells. The Western blot results indicated that PFKFB4 expression was increased in LNCaP-AI cells compared with LNCaP cells. Immunohistochemistry revealed that the expression of PFKFB4 in PCa tissue specimens was higher than that in BPH and adjacent tissue specimens. However, the differences in PCa tissue before and after ADT were not statistically significant.

**Conclusion:**

PFKFB4 may be associated with enhanced glycolysis during the androgen-independent growth of PCa cells in vitro. PFKFB4 may be a marker of PCa progression. Our results provide a rationale for further clinical investigation of PCa treatment focused on controlling PFKFB4 expression.

## Background

PCa has ranked as the leading cause of cancer-related death in males [1]. The PCa mortality rate in China has increased significantly in recent decades [2]. Hormonal therapy is currently the first-line therapy for advanced PCa and metastatic PCa; however, hormone-sensitive PCa cells inevitably become castration-resistant after a certain period of hormonal therapy [3, 4]. To our knowledge, the exact molecular mechanisms underlying the development of castration-resistant prostate cancer (CRPC) remain unclear. Previous research demonstrated that androgen-independent PCa cells could be created by blocking exogenous androgen in the culture medium.

Tumor cells depend on glycolysis for ATP generation, and targeting this dependence may represent an effective method for killing cancer cells, particularly cells with mitochondrial respiratory defects [5]. Regarding the treatment of CRPC, currently, enzalutamide and abiraterone are used to interrupt androgen receptor (AR) signaling and inhibit androgen synthesis in tumors, but PCa almost always becomes resistant after an initial period of response, and this phenomenon is often associated with increased glycolysis in tumor cells [5, 6]. Therefore, the regulatory proteins in glycolysis might be potential targets in cancer control.

PFKFB4 functions as a central control protein that regulates glycolysis in PCa cells [7]. Increasing evidence suggests that PFKFB4 plays important roles in the growth and progression of PCa. Our previous research indicated that silencing PFKFB4 could promote apoptosis in PCa and inhibit prostate tumor growth in vivo [3, 8]. The analysis indicated that the antagonists of PFKFB4 are possible therapeutic targets for PCa treatment.

LNCaP is a well-known androgen-sensitive PCa cell line [9], LNCaP-AI is an androgen-independent cell line that was created from LNCaP cells in a hormone-reducing environment [4, 10]. According to the abovementioned results, LNCaP-AI cells may be associated with increased glycolysis. To further understand the development of CRPC, LNCaP-AI cells should be the most helpful cell line, and this cell line has been widely used in the study of the molecular signaling pathways of CRPC [10, 11]. The present study aimed to observe the changes in glucose metabolism and PFKFB4 expression occurring during the androgen-independent growth of PCa cells. Based on the above research, the correlation of PFKFB4 expression with human tissue type was further evaluated, as this has rarely been reported in the published literature.

## Methods

### LNCaP cells and LNCaP-AI cell culture

LNCaP cells acquired from the Cell Bank of the Shanghai Institute of Biochemistry and Cell Biology, Chinese Academy of Sciences were cultured in RPMI-1640 medium (Gibco) supplemented with 10% fetal bovine serum (FBS; Gibco) at 37 °C in 5% CO_2_. LNCaP-AI cells were obtained according to the protocol reported in a previously published paper [4, 10]. Briefly, LNCaP-AI cells were cultivated in RPMI-1640 medium supplemented with 10% charcoal/dextran-stripped FBS (DCC-FBS; BioInd).

### Cell proliferation

Cell proliferation was measured using a cell counting kit-8 (CCK-8) assay. LNCaP and LNCaP-AI cells were harvested by exposure to 0.25% trypsin and seeded in a 96-well plate at a density of 0.5 × 10^4^ cells/well. The cells were incubated in medium with or without androgen for 1, 3, 5, or 7d. At the end of the incubation period, CCK-8 was added to each well and incubated at 37 °C for 100 min in a humidified 5% CO_2_ atmosphere. The absorbance at 450 nm of each well was read by a Multiskan (Thermo). Using time as the abscissa and the optical density at 450 nm (OD450) as the ordinate, cell growth curves were plotted.

### Total secreted PSA level measurement

LNCaP and LNCaP-AI cells were cultured in phenol-free RPMI-1640 medium supplemented with 10% DCC-FBS. The cells were maintained in medium without androgen for 2, 4, or 6d, and then medium supernatants were collected at the indicated time points for total PSA level measurement using the PSA-ELISA Kit (MultiSciences). The PSA secretion curves of cells were plotted by using OD450 values.

### Cell glucose consumption

LNCaP and LNCaP-AI cells were cultured in a 96-well plate at 1.2 × 10^6^ cells/well and incubated in RPMI-1640 medium supplemented with 10% FBS or phenol-free RPMI-1640 medium supplemented with 10% DCC-FBS, respectively. Both media contained a low concentration of glucose and penicillin-streptomycin. Medium supernatants and blank medium were collected, and the absorbance was measured after 18 h of culture. The glucose and lactate concentrations in medium samples were calculated by measuring the absorbance following the manufacturer’s instructions (Nanjing Jiancheng Bioengineering Institute). The difference between the value of the blank medium and a medium supernatant was used to calculate the amount of glucose consumption or lactic acid production.

### Western blot analysis

Cells were lysed on ice in RIPA lysis buffer (Beyotime) containing a protease inhibitor cocktail, the cell lysates were broken by hyper acoustic and centrifuged, and the supernatants were collected. Proteins were diluted in a gradient series, and protein concentrations measured using a Pierce BCA protein assay kit (MultiSciences). Then, the proteins were mixed with loading buffer, boiled for 5 min, separated by 10–12% SDS-PAGE, and transferred to 0.45-μm polyvinylidene difluoride membranes (Millipore) by electroblotting. The membranes were blocked for 1 h at room temperature and then incubated with a primary antibody diluted according to the manufacturer’s instructions. The membranes were washed 3 times for 5 min each time with TBST and incubated with HRP-conjugated secondary antibodies for 2 h at room temperature. The membranes were visualized using an ECL Western blotting detection system.

### Prostate tissue and pathological evaluation

Patients who underwent radical prostatectomy after receiving hormonal therapy in The People’s Hospital of Guangxi Zhuang Autonomous Region between January 2012 and February 2019 were recruited. BPH patients were used as the control group. The prostate tissues studied included PCa tissue specimens (biopsy and radical specimens), specimens of tissue adjacent to the carcinoma and BPH tissue specimens. Adjacent tissue was defined as benign tissue adjacent to cancerous tissue, as determined by a pathologist.

### Immunohistochemical staining and evaluation

Slides were subjected to immunohistochemical staining for PFKFB4 using a three-step method. PFKFB4 was detected using a rabbit anti-PFKFB4 polyclonal antibody (1:100; Abcam). Negative controls were generated using phosphate-buffered saline instead of the primary antibody. Positive controls were established following the instructions of the manufacturer. The staining results were blindly analyzed by experienced pathologists. The immunohistochemical results for PFKFB4 were categorized into four grades based on the staining intensity of positive cells.

### Statistical analysis

The proliferation of LNCaP and LNCaP-AI cells was compared by repeated-measure ANOVA*.* Glucose consumption and lactic acid production were analyzed by ANOVA. The Fisher’s exact test was used to compare the difference of PFKFB4 expressions in PCa tissue and BPH tissue. All statistical analyses were performed using IBM-SPSS v.24. *p* < 0.05 was considered statistically significant.

## Results

### Establishment and validation of LNCaP-AI cells

**Fig. 1:**
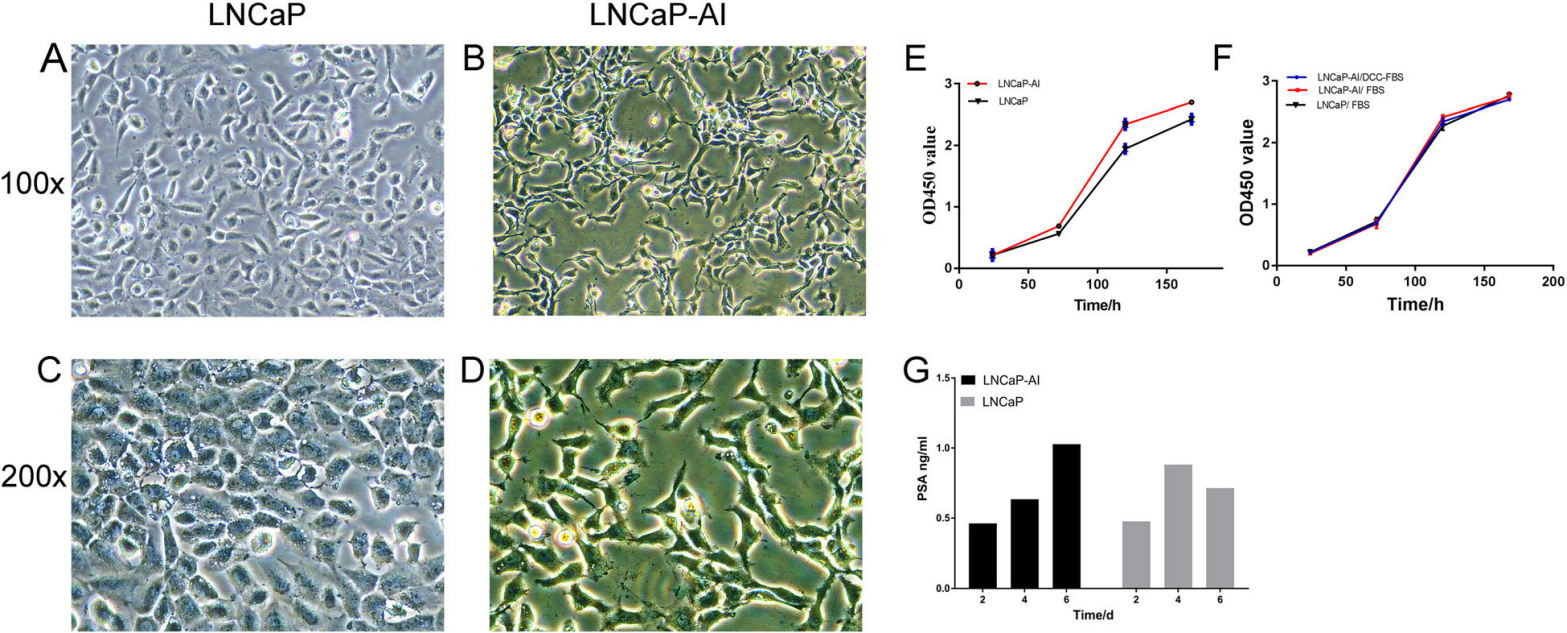
Morphological and biological characteristic differences between LNCaP and LNCaP-AI cells. Morphological: **a**, **c** The initial morphology of LNCaP cells before culture was a large cell body and short synapses; **b**, **d** The NE phenotype of LNCaP-AI cells exhibited shrinkage and rounding of the cell body and long synapses; the synapses wove a net between cells (magnification, 100x and 200x). Biological: **e** LNCaP-AI cell proliferation was significantly higher than that of LNCaP cells in androgen-free medium (*p* = 0.001). **f** The proliferation of LNCaP-AI cells in androgen-free medium was similar to that of LNCaP-AI and LNCaP cells in androgen-containing medium (*P* = 0.419). **g** In the hormone-free environment, LNCaP-AI cell PSA secretion was not affected, but LNCaP cells were significantly inhibited on day 6. Bars represent the mean ± SD of 3 replicates

To mimic the process of involved in the conversion to castration-resistant disease and following the methods of a previous study, we established an androgen-independent LNCaP-AI cell line derived from LNCaP cells cultured in RPMI-1640 medium containing 10% DCC-FBS. The initial morphology of LNCaP cells presented as a large cell body and short synapses (Fig. 1a and c). During 6 months of culture, some LNCaP cells underwent apoptosis, but the majority of cells developed an alternative autocrine mechanism through a series of morphological changes [12, 13]. After 3 weeks of culture, the results indicated that a multitude of LNCaP-AI cells had obvious synapses and were intertwined together as a web, which was distinct from the morphology and behavior of LNCaP cells (Fig. 1b and d). These morphological changes have also been found by other researchers [4], and this phenomenon might be one of the features of LNCaP-AI cells. To further explore the differences in biological characteristics between the LNCaP and LNCaP-AI cell lines, we compared cell proliferation and PSA secretion between the cells and found that LNCaP-AI cells proliferated much more than LNCaP cells (*p* = 0.001 Fig. 1e). The above findings suggested that LNCaP cells had converted into androgen-independent cells. The propagation of LNCaP-AI cells cultured in medium supplemented with 10% DCC-FBS was similar to that of LNCaP and LNCaP-AI cells cultivated in medium supplemented with 10% FBS (Fig. 1f, *P* = 0.419). In addition, LNCaP-AI cells maintained PSA secretion over time; however, LNCaP cells exhibited significantly inhibited secretion on day 6 in the same environment (Fig. 1g), which suggested that LNCaP-AI cells maintained the ability to secrete PSA better than LNCaP cells in the hormone-free environment.

### Increased glucose consumption and PFKFB4 expression in LNCaP-AI cells

**Fig. 2:**
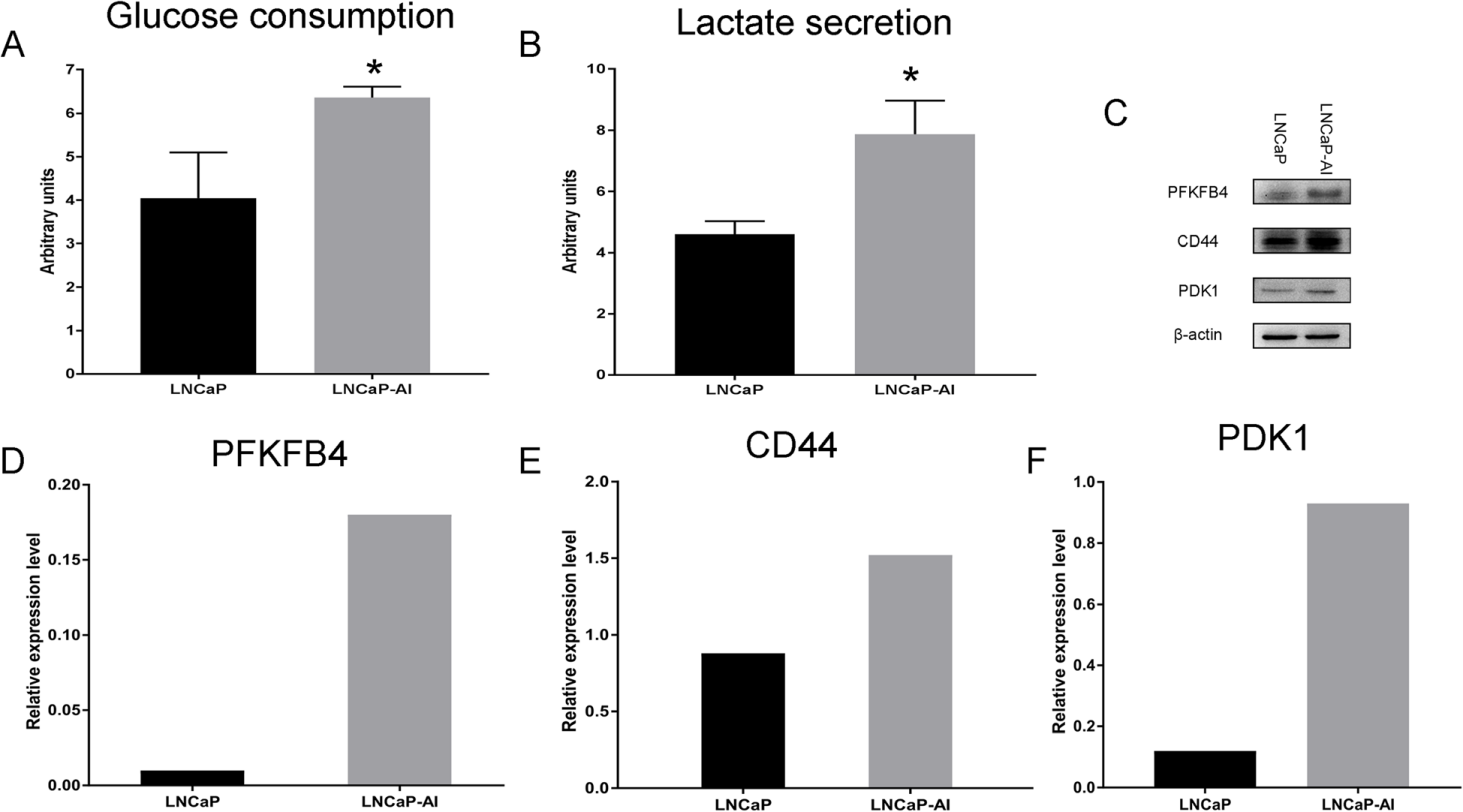
Increased glycolytic features and relative regulatory protein expression in LNCaP-AI cells compared with LNCaP cells. **a** Glucose consumption by LNCaP and LNCaP-AI cells (*, *P* = 0.02). **b** Lactate secretion by LNCaP and LNCaP-AI cells (*, *P* = 0.009). Bars represent the mean ± SD of 3 replicates. **c**, **d**, **e**, **f** Western blot detection of PFKFB4, CD44 and PDK1 expression in LNCaP-AI and LNCaP cells. Significantly increased expression of PFKFB4 (**d**), CD44 (**e**), and PDK1 (**f**) in LNCaP-AI cells compared with LNCaP cells

The conversion of LNCaP cells into LNCaP-AI cells was similar to the pathogenesis of CRPC. The glycolytic environment has been shown to regulate the response to therapy in CRPC cells [14, 15]. Our published research has demonstrated that androgen-independent PC-3 cells consume more glucose than androgen-dependent LNCaP cells, which is related to proliferation and aggressiveness. We thus speculate that glucose metabolism should be increasing during androgen-independent growth. Therefore, the glucose consumption and lactate production of LNCaP and LNCaP-AI cells were determined, and we found that the glucose consumption and lactate secretion of LNCaP-AI cells were significantly higher than those of LNCaP cells (Fig. 2a and b, *p* = 0.02 and *p* = 0.009, respectively).

Our previous studies have shown that CD44 may regulate glucose metabolism and reactive oxygen species (ROS) in PCa cells by the key enzymes of glucose metabolism, PFKFB4 and 3-phosphoinositide-dependent kinase-1 (PDK1). CD44 and PFKFB4 are highly expressed in androgen-independent cells [8], and glucose consumption is increased during androgen-independent growth. We therefore examined whether CD44, PFKFB4 and PDK1 are involved in androgen-independent growth in vitro. We next detected the expression of the abovementioned proteins in two different cell lines, LNCaP-AI and LNCaP. Western blot results indicated that the expression of CD44, PFKFB4, and PDK1 was increased in LNCaP-AI cells compared with LNCaP cells (Fig. 2c, d, e, and f).

### Expression of PFKFB4 in human PCa tissue

**Fig. 3:**
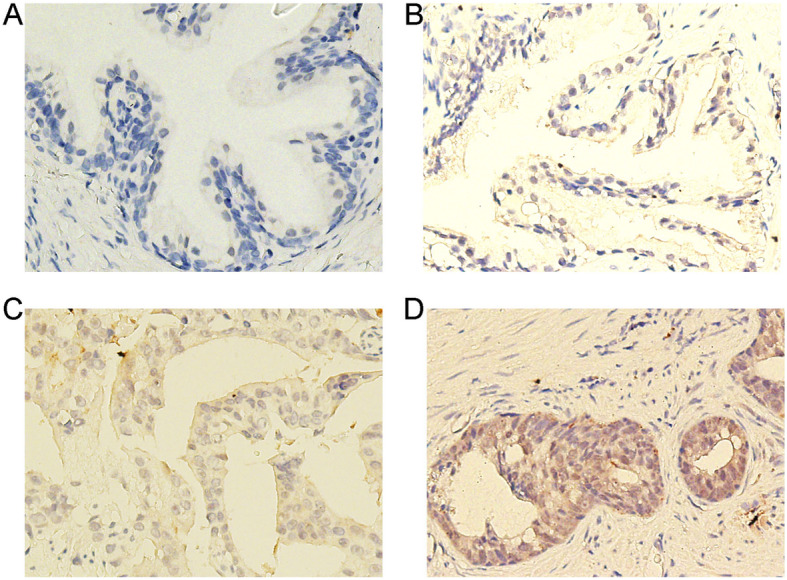
Immunohistochemical comparison of PFKFB4 expression between prostate cancer and BPH (magnification, 200X). **a** Staining for PFKFB4 in benign prostate tissue was negative. The positive staining for PFKFB4 appeared brown in prostate cancer samples, and the intensities were (**b**), weak; **c**, moderate; and (**d**), strong. Positive staining for the PFKFB4 protein was mainly found in the cytoplasm of PCa cells, and most intra- or extratumoral stromal cells were negative for PFKFB4

Cancer cells rely on glycolysis for energy, which may represent an available target for killing cancer cells or blocking drug resistance. PFKFB4, which has attracted more attention owing to its potential applications as a therapeutic target, is the major PFK2 isozyme in human cancers [16]. We therefore investigated the difference in PFKFB4 expression before and after endocrine therapy and the expression in BPH. In total, 25 patients with PCa who were treated with chemical castration before surgery were included in this research. Positive staining for the PFKFB4 protein was detected mainly in the cytoplasm of PCa cells, and most intra- or extratumoral stromal cells were negative [17]. PFKFB4 expression differed between PCa and BPH tissue samples. PFKFB4 expression was negative in BPH tissue specimens (Fig. 3a). The PFKFB4 expression intensity in tumor samples is shown in Fig. 3b, c and d. The staining score and statistical evaluation revealed that PFKFB4 expression in benign prostate tissue was lower than that in tissue samples collected before or after endocrine therapy (Table 1, *p* = 0.000). The expression of PFKFB4 differed significantly between PCa and adjacent tissue samples (Table 1, *p* = 0.001). However, the difference in PCa tissue samples collected before and after endocrine therapy was not statistically significant (Table 1, *p* = 0.377).

**Table 1:** Comparison of the expression of PFKFB4 among different types of PCa tissue and BPH tissue

Group		PFKFB4 expression		Number (n)	Total (n)	*P*-value^*^
		Negative (n)	Positive (n)			
PCa^a^ and BPH	PCa^a^	7	18	25	40	0.000
	BPH	15	0	15		
PCa^b^ and adjacent tissue	PCa^b^	11	14	25	50	0.001
	Adjacent tissue	23	2	25		
Before and after therapy^c^	Before therapy^d^	11	14	25	50	0.377
	After therapy^e^	7	18	25		

^a, e^Radical prostatectomy tissue samples; ^b, d^preoperative biopsy tissue; ^c^biopsy tissue collected before hormonal therapy and surgical tissue collected after hormonal therapy, with both samples derived from the same patient. ^*^Based on the Fisher’s exact test

## Discussion

In the present study, we successfully established LNCaP-AI cells by following the methods of a previous report. The characteristics of the ideal model for PCa cells are androgen dependence and wild-type AR expression. Moreover, these cells should also have the ability to convert into androgen-independent cells in vitro. Currently, common PCa cell lines include LNCaP, PC-3, DU-145, and C4–2 derived from different tissues. PC-3, DU-145, and C4–2 are all androgen-independent cell lines, and the markers of the above cell lines also differ [18–20]. The evolution of these androgen-independent cell lines is discontinuous, so it is difficult to draw a reliable conclusion from the comparison of these androgen-independent cell lines. Therefore, these cell lines are not the most ideal cell lines for castration-resistant growth research. LNCaP is an androgen-dependent cell line that is widely used in various experiments. LNCaP cells show the differentiation characteristics of primary PCa, including androgen-dependent growth and the ability to secrete PSA in vitro. In this study, charcoal/dextran [21] was used to remove most androgen from the culture medium, which mimicked the clinical process of androgen deficiency treatment. Therefore, compared with PC-3, DU145, and C4–2 cells, LNCaP cells are a more reasonable and reliable choice for the study of castration resistance.

To confirm the establishment of LNCaP-AI cells, we observed changes in the cell morphology, proliferation rate and PSA levels of LNCaP cells. During the whole induction process, the features of neuroendocrine (NE) cells emerged as a hallmark of the conversion to androgen-independence (Fig. 1d). The NE phenotype is widely accepted to be the most prominent characteristic of LNCaP-AI cells and promotes aggressive phenotypic transformation [13, 22]. CCK-8 analysis confirmed that LNCaP-AI cells could adapt to the androgen-depleted environment (Fig. 1f). LNCaP-AI cells proliferated more rapid than LNCaP in the same medium (Fig. 1e). ELISA confirmed that the PSA secretion of LNCaP-AI cells rose gradually and exceeded that of LNCaP cells on the 6th day. But in first 6 days, the proliferation rate of LNCaP-AI was similar to that of LNCaP, and the PSA levels was lower than that of LNCaP cells. Previous studies showed that the PSA secretion of LNCaP-AI cells was greater than that of LNCaP cells [23]. The reason for this difference may be that charcoal/dextran can’t completely strip hormones in FBS [21]. LNCaP cells can continue to use the remaining androgen to maintain the above biological behavior. Another possible reason is that LNCaP-AI cells appear to be stem cell-like cells with the feature of a low PSA level and the morphology of androgen-independent cells [22, 24]. Proliferation rate and PSA levels gradually increased when LNCaP-AI cells were continuously differentiated. In general, the establishment of LNCaP-AI cells is a continuous process that provides a good model for studying the mechanism underlying CRPC development in vitro.

It is well known that the enhanced proliferation of cancer cells is due to cancer cells having higher glycolytic activity than benign cells, which increases glucose uptake and lactate secretion. Our results indicated that the glucose consumption and lactate secretion of LNCaP-AI cells were increased after androgen-independent growth. This result is the same as the finding of our previous study, which indicated that androgen-independent PC-3 cells had higher glycolytic levels than androgen-dependent LNCaP cells [3]. In this study, we further demonstrated that glucose metabolism might be an important difference between two tumor types and therefore might be a potential target for the treatment of CRPC. Evaluation of the change in glycolytic metabolism during androgen-independent growth is a relatively good mimic of the glycolytic process occurring in CRPC and could provide the metabolic basis for the study of the unique biological behaviors of CRPC.

Importantly, tumor metabolism is extremely complex. Cancer cells undergo energy metabolism reprogramming to adapt to the changing microenvironment [25], including changes to glycolytic metabolism, transcriptional regulation and signal transduction [26]. These changes require the involvement of enzymes. Based on current research on PFKFB4, this enzyme regulates glucose metabolism in tumor cells, which has been widely recognized. PFKFB4 controls glycolysis and the pentose phosphate pathway (PPP) by regulating F-2,6-BP. ATP and lactic acid production in glycolysis are associated with proliferation and drug resistance in tumor cells. NADPH in the PPP is associated with stabilizing redox homeostasis and lipid synthesis in tumor cells, which are associated with tumor cell survival [7, 16]. Our previous research demonstrated that PFKFB4 was highly expressed in androgen-independent cells DU145 and PC-3 cells compared with androgen-dependent LNCaP cells and could regulate the proliferation, invasion, and migration of PC-3 cells [3, 8]. In the present study, PFKFB4 expression increased significantly with androgen-independent growth, suggesting that PFKFB4 is involved in the behavior of PCa cells during the phenotypic switch from adenocarcinoma to CRPC. Therefore, based on the consensus on PFKFB4 and our previous studies, we consider that this phenomenon likely occurs through the regulation of glucose metabolism. This speculation is not without foundation. The findings from another study showed that the expression of PFKFB4 was significantly higher in small cell neuroendocrine carcinoma than in adenocarcinoma, which suggested that increasingly aggressive PCa might be related to increasingly high PFKFB4 expression [3]. Moreover, the expression of PFKFB4 is higher in metastatic PCa than in primary lesions [7]. However, PFKFB4 was also involved in tumor progression through redox homeostasis, transcriptional regulation, autophagy and so on [16]. Therefore, PFKFB4 was related to androgen-independent growth and might be a potential therapeutic target for CRPC treatment. We had a preliminary understanding of the role of PFKFB4 in androgen-independent state, so the next step was to determine association and mechanism between PFKFB4 and proliferation and migration of androgen independent cells, furthermore, the role of PFKFB4 as glycolytic protein in androgen independent growth.

Western blotting also showed that the expression of CD44 and PDK1 increased when LNCaP cells transformed into LNCaP-AI cells, which might be involved in the high glycolytic activity accompanying PFKFB4 expression. PDK1 and CD44 are regulatory enzymes in glucose metabolism [3, 8]. PDK1 can promote lactate production in tumor cells by increasing glycolysis and making the microenvironment acidic, which increases the invasive behavior of malignant cells [27]. PDK1 also regulated the proliferation, invasion, and migration of PC-3 cells in our previous studies, which was similar to the results for PDK1-regulated androgen-independent growth found in this study. CD44 is a multifunctional enzyme. CD44 promotes antioxidant activity and drug resistance in cancer cells by modulating glucose metabolism [28]. We have identified that CD44 regulates glycolysis in PC-3 and LNCaP cells. Research has shown that CD44 is expressed in PC-3 cells but not in LNCaP cells [29]. However, according to our Western blot results, CD44 was expressed in LNCaP-AI and LNCaP cells. The possible reason is that CD44 expression is associated with cells of the NE phenotype in human PCa cell lines [30]. LNCaP cells exhibited NE features in our results. Our laboratory has established that CD44 regulates PDK1 and PFKFB4 expression in PCa cells, PDK1 and PFKFB4 expression is higher in LNCaP-AI cells than in LNCaP cells, and glucose consumption is increased during androgen-independent growth. Therefore, the results of the present study suggest that CD44 may be involved in the androgen-independent transformation of LNCaP-AI cells through the upregulation of glucose metabolism by PFKFB4 and PDK1.

The immunohistochemical staining results showed that the staining intensity of PCa tissue was significantly stronger than that of BPH tissue. It is consistent with our previous research results, the intensity of PFKFB4 expression is proportional to the type of PCa [3]. It was known ADT could induce the CRPC [31], based on these findings and our results, it was reasonable that PFKFB4 expression would increase accompany with ADT. However, the staining of ADT tissue in present study showed no statistically difference before and after ADT. The main reason was the duration of neoadjuvant ADT, usually no more than 3 months. In hormone-sensitive stage, ADT inhibited proliferation and glycolytic activity of cancer cells which potentially inhibited PFKFB4 as well. As we all know, development of CRPC in ADT-treated tumor can take years to emerge clinically [3]. So, after short term neoadjuvant ADT, tumor cells were also hormone-sensitive, which could explain the findings of immunohistochemical staining. Taken together, high expression of PFKFB4 was the hallmark of tumor proliferation and androgen-independent growth, which suggested PFKFB4 might be a novel biomarker in prostate cancer.

There are several possible limitations to this study. We performed only glucose consumption and lactate secretion assays for metabolic evaluation. The glucose metabolism and PFKFB4 expression of LNCaP-AI cells need to be compared with those of other androgen-independent cell lines. The PFKFB4-focused in vivo experiments with LNCaP-AI cells need further improvement. The human PCa tissue sample size needs to be enlarged, and the expression pattern of PFKFB4 in CRPC tissue remains unclear.

## Conclusions

The experimental results show that the LNCaP-AI cell model successfully simulates the clinical process of CRPC transformation in vitro and that glucose metabolism and PFKFB4 expression are increased when PCa cells develop the androgen-independent phenotype. In addition, we further confirmed the relationship among CD44, PFKFB4, and PDK1. PFKFB4 is associated with androgen-independent growth of PCa cells in vitro which may by increasing glycolysis. However, future trials involving knockdown of the expression of glycolysis-regulating enzymes in LNCaP-AI cells should be performed to confirm these preliminary findings. PFKFB4 is highly expressed in human PCa tissue, and the expression intensity of PFKFB4 is related to the type of PCa; PFKFB4 may be a marker of PCa progression. In conclusion, our results and previous mechanistic studies could advance the understanding of the level of glucose metabolism and the expression of glucose metabolism-regulating enzymes in CRPC, which provide a rationale for further clinical investigation of tumor treatment focused on controlling PFKFB4 expression.

## Data Availability

The datasets used and/or analyzed during the current study are available from the corresponding author on reasonable request.

## Abbreviations

ADT: Androgen-deprivation therapy
PCa: Prostate cancer
PFKFB4: 6-phosphofructo-2-kinase/fructose-2,6-biphosphatase 4
LNCaP-AI: Androgen-independent LNCaP
BPH: Benign prostatic hyperplasia
CRPC: Castration-resistant prostate cancer
AR: Androgen receptor
FBS: Fetal bovine serum
DCC-FBS: charcoal/dextran-stripped fetal bovine serum
ROS: Reactive oxygen species
CCK-8: Cell counting kit-8
PDK1: 3-phosphoinoside-dependent kinase-1
NE: Neuroendocrine
PPP: Pentose phosphate pathway
